# Microcalcifications Detected as an Abnormality on Screening Mammography: Outcomes and Followup over a Five-Year Period

**DOI:** 10.1155/2013/458540

**Published:** 2013-10-01

**Authors:** Melissa Craft, Anne M. Bicknell, Georges J. Hazan, Karen M. Flegg

**Affiliations:** ^1^Department of Medical Imaging, The Canberra Hospital, Woden, Canberra, ACT 2606, Australia; ^2^Breastscreen ACT, Canberra, ACT 2600, Australia; ^3^Australian National University Medical School, Woden, Canberra, ACT 2606, Australia

## Abstract

*Objectives*. This study reviewed the outcome of women attending a breast screening program recalled for assessment of microcalcifications and examined the incidence of a breast carcinoma detected during the following five years in any of the women who were given a benign diagnosis at assessment. *Method*. A retrospective study consisted of 235 clients attending an Australian BreastScreen program in 2003, who were recalled for investigation of microcalcifications detected on screening mammography. Records for the following five years were available for 168 women in the benign outcome group including those who did not require biopsy at initial assessment. *Results*. Malignant disease was detected in 26.0% (*n* = 146) of the women who underwent biopsy. None of the women in the benign outcome group, with available five-year follow-up records, developed a subsequent breast cancer, arising from the calcifications initially recalled in 2003. *Conclusions*. This study highlights the effectiveness of an Australian screening program in diagnosing malignancy in women with screen detected microcalcification. This has been achieved by correctly determining 38% (*n* = 235) of the women as benign without the need for biopsy or early recall. A low rate of open surgical biopsies was performed with no cancer diagnoses missed at the time of initial assessment.

## 1. Introduction

The BreastScreen Australia Program screens over 1.5 million women for breast cancer throughout Australia every two years [1]. During the period 1993–2011, BreastScreen Australian Capital Territory and South East New South Wales (BreastScreen ACT & SENSW) offered a free screening mammography service to all female Australian citizens residing in the ACT and South East NSW. Though the service is targeted at women aged 50–69, women aged 40–49 years and older than 69 years were screened at their request. Screening involves two mammographic views of each breast: craniocaudal and mediolateral oblique.

Abnormalities detected on screening mammography are categorised according to the imaging classifications (1–5) recommended by the *National Breast Cancer Centre Breast Imaging Report* [2]. The five breast imaging classifications are category 1, no significant abnormality; category 2, benign; category 3, indeterminate/equivocal; category 4, suspicious for malignancy; and category 5, radiologically malignant. At BreastScreen ACT & SENSW, a breast imaging classification 1 or 2 on screening mammogram is considered benign and results in the client being placed on recall for routine screening. A breast imaging classification 3, 4, or 5 on screening mammography leads to recall of the client for assessment. Workup at the assessment clinic may include clinical examination, further mammographic views with reassignment of imaging category, ultrasound, fine needle aspirate (FNA), or core needle biopsy (CNB). CNB is performed either under ultrasound guidance (UCNB) 14–16 gauge or as vacuum assisted large core stereotactic biopsy (VALCS) 11 gauge. An open surgical biopsy may be required in some circumstances. 

The program complies with the National Accreditation Standards, set by BreastScreen Australia, of recalling for further assessment for a maximum of 10% of women in their first screening episode and 5% of women attending for their second or subsequent screening round, to minimise unnecessary investigations in women screened.

The purpose of this study was to determine the incidence of malignancy detected with further assessment of women with indeterminate, suspicious, or malignant calcification (breast imaging classifications 3, 4, and 5) identified on their screening mammogram. Of all calcifications categorised as indeterminate, suspicious, or malignant, the incidence of associated malignancy has been reported by previous studies by approximately 10%–48% [3–12]. While most studies report a malignancy rate of 20%–30%, a recent study by Farshid et al. [3] gives a figure of 47.9%. 

## 2. Methods

The year 2003 was chosen as it provided an opportunity for five years of followup. All clients of BreastScreen ACT & SENSW who had calcification reported as imaging classification 3, 4, or 5 on screening mammogram and who were recalled for workup were included. A total of 235 eligible women were identified, and their medical records were reviewed retrospectively. The mammography images performed in 2003 were analogue screen film. The cohort included both women who were recalled for calcifications alone and those who had a density associated with calcifications. 

For each case, the following data was extracted from clinical records: patient age, the breast imaging classification as revised at assessment, ultrasound findings (if performed at assessment), all investigations that were conducted, the diagnostic outcome of the assessment, and any upgrade or downgrading of pathology at surgical excision during treatment was noted. In addition, the size of the lesion was measured in millimetres from the nonmagnified mammographic view demonstrating the largest diameter. The original mammograms were unable to be obtained for 40 of the 235 women, and thus these women were excluded from this part of the analysis. The size in millimetres of malignancy from surgical pathology at treatment was noted and nodal metastases in cases of invasive carcinoma were included in the data. The medical records for 168 women in the benign group were reviewed from 2003 to 2005 in order to note the development of breast malignancy occurring either through diagnosis at a subsequent screening episode or as an interval cancer between screening intervals. Notification of interval cancers at BreastScreen is obtained by direct advice from clients, surgeons, and general practitioners and is also formally requested from the cancer registry. 

 If a diagnosis of malignancy occurred after 2003 and before April 2008, the location and nature of the malignancy was compared to the location of the calcifications that lead to inclusion in this study. All imaging and pathology performed at BreastScreen ACT & SENSW assessment clinic have been reviewed by two radiologists and two pathologists as a standard quality assurance procedure. For the purpose of this study, a third radiologist independently reviewed the imaging and pathology of any woman who was diagnosed with a malignancy in the ipsilateral breast during the five-year follow-up period. 

Cases were stratified into four groups according to the assessment outcome.

(1)  Benign: lesions diagnosed as benign with and without biopsy (this includes lesions diagnosed as benign with a pathological confirmation including fibroadenoma, fibrocystic change, sclerosing adenosis, sclerosing papilloma, and ductal hyperplasia with no atypia following FNA, UCNB, VALCS, or open surgical biopsy).
(2)  Atypical: borderline lesions diagnosed as atypical following FNA, UCNB, VALCS, or open surgical biopsy, including atypical ductal hyperplasia (ADH), and atypical lobular hyperplasia (ALH). In 2003 (the year of the study), women with a core biopsy showing atypical lobular hyperplasia at BreastScreen ACT were considered as having a high risk lesion requiring annual routine screening without the need for surgical excision biopsy. This policy has changed over subsequent years.
 There were no cases of other borderline lesions such as lobular carcinoma in situ (LCIS) or atypical flat epithelial lesions.
(3)  Ductal carcinoma in situ: lesion diagnosed as noninvasive malignancy following FNA, UCNB, VALCS, or open surgical biopsy (DCIS).
(4)  Invasive carcinoma: lesion diagnosed as invasive malignancy, following FNA, UCNB, VALCS, or open surgical biopsy.

For women with a diagnosis of DCIS or invasive carcinoma following FNA, UCNB VALCS, or surgical open biopsy from assessment, the pathology from surgical excision at treatment was reviewed for upgrading or downgrading of the lesion and this diagnosis was considered the final diagnostic outcome. Both the size of invasive carcinoma following surgical excision and presence of nodal metastases were recorded.

For the purpose of statistical analysis, benign and atypical cases were grouped and denoted benign, and in situ carcinoma and invasive carcinoma were grouped and denoted malignant. Statistical differences between groups were analysed by two-sided chi-squared tests and *P* values of less than 0.05 were considered significant. 

This study was approved by the ACT Department of Health Ethics Committee and the Human Research Ethics Committee of the Australian National University.

## 3. Results

A total of 235 women recalled for assessment of calcifications categorised with a breast imaging classification 3, 4, or 5 on screening mammogram at BreastScreen ACT & SENSW, in 2003. The distribution of clients age and the outcome of assessment are shown in Table 1.

The majority of lesions were deemed to be completely benign (81.3%, *n* = 191). However, 38 women (16.2%) were found to have malignant lesions with 47.4% (*n* = 18) of these being invasive and 52.6% (*n* = 20) of these being ductal carcinoma in situ (DCIS). A mammographic density was associated with the calcifications in 26 of the recalled women. The majority of these women had a benign outcome from assessment (53.8%, *n* = 14), 38.5% (*n* = 10) had invasive carcinoma, and 7.7% had DCIS (*n* = 2). 

The distribution of diagnoses and the investigations performed at assessment are shown in Table 2.

Women whose eventual diagnosis was a malignancy were more likely to have undergone FNA (71.4% of FNAs compared to 28.6%) or UCNB (100% of UCNB). However, the majority of women who underwent VALCS had a benign outcome (76.8%). There was no significant difference between benign and malignant cases and the rates of open surgical biopsy. 

Table 3 shows the revised breast imaging classification attributed at assessment. Almost half (39.1%, *n* = 92) had a revised breast imaging classification of 1 or 2 and required no further investigations. Only women with a malignant outcome were given an imaging classification of 5 and such women contributed to 60.9% of classification 4. Women with a benign outcome contributed to 87.3% of classification 3 (as compared to malignant outcomes contributing 12.7%, *P* = 0.0001). 

The distribution of assessment diagnosis and the greatest diameter of the lesion of calcifications are demonstrated in Table 4. Of the benign group many more women were likely to have lesions of calcifications extending less than 11 mm in diameter (75.2%, *n* = 124 of 165), compared to 24.9% (*n* = 41 of 165) who had lesions 11 mm and over in size. Those in the malignant group were shown to have 46.7%, (*n* = 14 of 30) with calcifications extending less than 11 mm in diameter and 53.3% (*n* = 16 of 30) had calcifications extending >10 mm. 

Of the 191 women reported as benign and the six women given an atypical result, 168 women had future screening information available (29 women had no future information). Of these 168 women with future screening information, five (3.0%) had a malignancy detected during the five-year follow-up period (2003–2008). One woman was recalled after screening, in 2007, with a stellate lesion adjacent to the calcifications in the ipsilateral breast that lead to inclusion in this study. The stellate lesion was shown to be invasive ductal carcinoma Grade 2 with associated noncalcified DCIS present. The adjacent calcifications had not been biopsied during the 2003 assessment; however, further review during the second visit, in 2007, confirmed that these calcifications were consistent with benign fibrocystic disease. In addition, one woman was found to have an invasive ductal carcinoma Grade 3 with DCIS high nuclear grade in a new cluster of calcifications arising superoposterior to the calcifications for which she was recalled, in 2003. Subsequent biopsy of these previously assessed calcifications showed benign stromal calcification and the presence of incidental noncalcified low grade DCIS. The remaining three cases of malignancy were shown to be at sites in the contralateral breast unrelated to the calcifications that lead to inclusion in this study. 

## 4. Discussion

Calcification is one of the important features sought in screening mammography as a possible indicator of the presence of early breast carcinoma [13]. In this study, we examined the significance of calcifications detected on screening mammogram in the diagnosis of breast carcinoma at BreastScreen ACT & SENSW in 2003. 

The incidence of malignancy in all women with screen detected calcifications that required assessment was 16.1% (*n* = 38). This included women with and without biopsy confirmation of outcome. 

The incidence of malignancy in women with screened detected calcifications who underwent pathological diagnosis at assessment was 26.0% (*n* = 38) which is comparable to previous studies (20%–30%) [4, 7] although not as high as others [3, 10] who record values of up to 47.9% [3]. Our work differs from previous studies by including a five-year followup for subsequent development of malignancy of all women who were initially recalled for evaluation of microcalcification, including those who were given a benign diagnosis based on the imaging and clinical examination alone without the need for biopsy. This was done to check the validity of the outcomes of assessment. In addition, we determined if any of the subsequently diagnosed malignancies were at the same site as the calcifications assessed, in 2003. 

As previous studies have not included followup of women recalled who were given a benign outcome based on imaging and clinical examination alone without biopsy, it is not known if there may have been an increased rate of missed cancer diagnoses in women from these studies. The difference in malignancy rate may also have been influenced by the smaller sample size necessitated by the different design of our study. The rate of open surgical biopsy in our study is low at 6%. It is not clear in previous studies citing a higher malignancy rate whether there is also an increased rate of surgical open biopsy. 

Microcalcification is the most common mammographic feature of DCIS, occurring in 80%–90% of DCIS with mammographic abnormality [14]. Of all malignancies identified in this study, the incidence of in situ carcinoma (DCIS) 13.9% (*n* = 20) was higher than the incidence of invasive carcinoma 12.5% (*n* = 18). These data are consistent with the widely published view that calcifications are more likely to be associated with DCIS than invasive carcinoma on screening mammogram [13]. DCIS has become increasingly diagnosed since the advent of widespread mammographic screening and is an important entity due to its association with invasive malignancy [15]. The pathological diagnosis of DCIS is generally accompanied by an assessment of nuclear grade, assigned based on the morphological level of differentiation of the malignant cells. The nuclear grade of DCIS has been shown to predict the likelihood of recurrence and the progression of DCIS to invasive carcinoma [16]. Of all cases of DCIS, the largest proportion was pathologically diagnosed as high nuclear grade (50% *n* = 10), followed by intermediate nuclear grade (30%, *n* = 6), and finally low nuclear grade (20% *n* = 4). Previous studies have found a similar distribution in cases of DCIS [3, 15]. This is mainly due to the characteristics of high nuclear grade DCIS (such as reduced cellular differentiation and necrosis), which facilitate diagnosis of mammographic abnormalities, particularly calcifications [17].

Calcification has also been found to be associated with atypical breast lesions, which are borderline breast lesions that have uncertain malignant potential [18]. Of all final diagnoses following needle biopsy and surgical biopsy, six (2.6%) were atypical, with ALH and ADH contributing equal proportions.

 Benign lesions which included all cases determined benign on clinical examination and imaging alone and all cases pathologically diagnosed as benign made up the largest proportion of final outcomes (81.3%, *n* = 191). This is consistent with previous studies which indicate that the majority of microcalcifications will be benign but, due to their occasional association with malignancy, require investigation [19]. 

The investigations conducted during assessment of women with screen detected mammographic calcifications were found to vary with the eventual outcome of assessment. VALCS was the most commonly performed investigation, with the largest proportion of benign, atypical, and carcinoma in situ cases undergoing this investigation more than any other investigation. VALCS is considered a reliable alternative to open surgical biopsy in providing a histological diagnosis of calcifications with breast imaging classification 3, 4, or 5 [11, 20–23]. In addition, open surgical biopsy was rarely required (5.8%, *n* = 9). This indicates that VALCS is utilised as an effective alternative to open surgical biopsy in the assessment of calcifications in this screening program.

The breast imaging classification system utilised for reporting of mammographic breast abnormalities at BreastScreen ACT & SENSW is designed to provide standardised breast imaging terminology for categorising mammograms in screening centres in Australia. This system differs from the Breast Imaging Reporting and Data Systems (BI-RADS) used throughout North America in that it is a five-tiered grading system, rather than a seven-tiered system, and is designed to be applied to screening mammograms [24]. In this study, women that were assigned breast imaging classification 1 or 2 at assessment were considered benign and did not require FNA, UCNB, VALCS, or open surgical biopsy. Those assigned breast imaging classification 3, 4, or 5 underwent further investigation of some kind, most often VALCS. Only a small proportion of cases with a benign final outcome (4.6%, *n* = 9) were assigned breast imaging classification 4 and none assigned breast imaging classification 5. A significantly greater proportion of benign cases were assigned breast imaging classification category 3 than malignant cases (*P* = 0.0001). This study validates the breast imaging classification system used for reporting screening mammograms. Data showing the dimensions of the malignancy for women with invasive carcinoma showed that 76% had a cancer size less than or equal to 15 mm, highlighting the success of early detection of small cancers within the screening program. 

Two women in this study were subsequently found to have an invasive ductal carcinoma detected at sites adjacent to the calcifications that were determined benign following assessment in 2003. These two clients did not undergo biopsy in 2003 as the calcifications were regarded as benign (breast imaging classification category 2) following clinical examination and imaging alone. During their subsequent assessment (with the eventual malignant outcome), both women had the areas of calcification reviewed that had been assessed previously and it was shown that the original areas of calcification were not related to the subsequent malignancy. Two independent radiological opinions have been sought to confirm this impression. Three women developed malignancy in the contralateral breast during the five-year follow-up period. Thus, of the five (2.1%) women diagnosed with a breast malignancy at future screening including those who did not have a biopsy of the calcification at the initial visit, none were shown to have a carcinoma developed from the lesion that led to their inclusion in this study.

The extent, morphology, and distribution of calcifications can be used to predict their aetiology [13]. We compared the greatest diameter of the lesion of microcalcifications with the outcome of assessment. The benign cases were significantly more likely to have lesions of calcifications 0–5 mm in diameter than malignant cases (*P* < 0.0001). One previous study suggests that microcalcifications are more likely to be associated with malignancy if they extend over a greater area [10]. This was not the case in our study where the diameter of lesions of malignant calcifications was extremely varied in size. The morphology of the calcifications was not assessed in this study. This indicates another area of future research.

The main limitation of this study is that it only provides a small snapshot of information from one BreastScreen service in Australia, over one year. While the findings of this study may be generalised to other areas, further research incorporating a larger sample size by including other BreastScreen regional services would be useful. Another limitation of this study is the loss to followup of women. There were 29 women who did not attend screening after 2003. While this is only 12.3% of the total sample, it would be preferable to have five-year follow-up data on all women. The small geographical area covered by BreastScreen ACT & SENSW is advantageous to gaining follow-up information because if a client is treated for a breast carcinoma between screening intervals in this region, then our experience indicates that the BreastScreen program is usually notified by either the surgeon or general practitioner. The State and Territory Cancer Registry is also checked regularly by our screening program for women who may have developed interval cancers. 

## 5. Conclusion

Mammographic assessment of calcifications and classification according to the NBCC breast imaging classification is an essential part of assessment of potentially abnormal screening mammograms. The incidence of malignancy associated with a mammographic abnormality of microcalcification in our study is comparable to results shown by some investigators and less than other investigators. Sample size may play a role. It is not clear in previous studies whether the higher malignancy rates are associated with an increased rate of open surgical biopsy or missed cancer diagnosis at initial assessment.

This study differs from others in that the women recalled for further evaluation who had a benign outcome, including both those who had no biopsy and those who underwent biopsy diagnosis, have been reviewed for a five-year follow-up period. This study demonstrates that the assessment protocol based on limiting FNA, UCNB, VALCS, and open surgical biopsy to lesions with a breast imaging classification of 3, 4, or 5 is an effective strategy for all women recalled with calcifications. It also confirms that ductal carcinoma in situ lesions of high nuclear grade comprise a significant proportion of in situ carcinomas diagnosed through this breast screening program. The majority of invasive carcinomas detected were small at less than 15 mm shown on surgical treatment pathology. 

The study highlights the effectiveness of an Australian screening program in diagnosing malignancy in women recalled with screen detected microcalcification, and particularly in diagnosing small invasive cancers with no evidence of missed cancer diagnosis as determined by longitudinal followup. 

## Figures and Tables

**Table 1:** Age distribution and final diagnostic outcome* of clients with calcifications assigned a breast imaging classification 3, 4, or 5 on screening mammogram.

	*n* (%)	Total *n* (%)
Age (years)		
<50	18 (7.7)	235 (100.0)
50–59	123 (52.3)	
60–69	85 (36.2)	
≥70	9 (3.8)	
Final outcome		
Benign		
Benign	191 (81.3)^†^	191 (81.3)
Atypical		
ADH	3 (1.3)	6 (2.6)
ALH	3 (1.3)	
DCIS		
Low nuclear grade	4 (1.7)	20 (8.5)
Intermediate nuclear grade	6 (2.6)	
High nuclear grade	10 (4.3)	
Invasive carcinoma^‡^		
Grade 1^§^	7 (3.0)	18 (7.7)
Grade 2	5 (2.1)	
Grade 3^§^	6 (2.6)	

*Outcome determined at assessment which may include results of FNA, UCNB, VALCS, or open surgical biopsy or surgical treatment pathology whichever is the latter.

^†^89 cases (46.6% of the benign outcomes) did not undergo FNA, UCNB, VALCS, or open surgical biopsy.

^‡^Five of the 18 women with invasive carcinoma had nodal metastases (three with invasive carcinoma grade 3 had one metastatic node; one with invasive carcinoma grade 3 had 12 metastatic nodes; one with invasive carcinoma grade 1 had one metastatic node).

^§^includes one case upgraded from DCIS to invasive carcinoma as a result of surgery.

**Table 2:** Distribution of biopsy investigations conducted with final client outcome*.

Biopsy method performed^†^	Final outcome				Total of each biopsy method *n* = 156 (% of all biopsy methods)
	Benign *n* (% of each biopsy method)	Atypical *n* (% of each biopsy method)	In situ carcinoma *n* (% of each biopsy method)	Invasive carcinoma *n* (% of each biopsy method)	
FNA	2 (28.6)	0 (0)	0 (0)	5 (71.4)	7 (4.5)
UCNB	0 (0)	0 (0)	0 (0)	8 (100)	8 (5.1)
VALCS	101 (76.5)	3 (2.3)	20 (15.1)	8 (6.1)	132 (84.6)
Open surgical biopsy	1 (11.1)	3 (33.3)	4 (44.4)	1 (11.1)	9 (5.8)

*Outcome determined at assessment which may include results of FNA, UCNB, VALCS, or open surgical biopsy or surgical treatment pathology whichever is the latter.

^†^Investigations conducted at assessment were not mutually exclusive and some clients had more than one biopsy type.

**Table 3:** Distribution of revised breast imaging classification as a result of assessment, with final outcome*.

Revised breast imaging classification category^†^	Total of each imaging category *n* = 235 (%)	Benign outcome			Malignant outcome			Benign versus malignant^§^ *P* value
		Benign *n* (% of imaging category)	Atypical *n* (% of imaging category)	Total benign^§^ *n* = 197 (% of this imaging category)	In situ carcinoma *n* (% of imaging category)	Invasive carcinoma *n* (% of imaging category)	Total malignant^§^ *n* = 38 (% of this imaging category)	
1	6 (2.6)	6 (100)	0 (0)	6 (100)	0 (0)	0 (0)	0 (0)	0.0143
2	86 (36.6)	86 (100)	0 (0)	86 (100)	0 (0)	0 (0)	0 (0)	0.0001
3	110 (46.8)	91 (82.7)	5 (4.6)	96 (87.3)	11 (10)	3 (2.7)	14 (12.7)	0.0001
4	23 (9.8)	8 (34.8)	1 (4.3)	9 (39.1)	7 (30.4)	7 (30.4)	14 (60.9)	0.2971
5	10 (4.2)	0 (0)	0 (0)	0 (0)	2 (20)	8 (80)	10 (100)	0.0016

*Outcome determined at assessment which may include results of FNA, UCNB, VALCS, or open surgical biopsy or surgical treatment pathology whichever is the latter.

^†^Clients were assigned a new breast imaging classification at assessment following further mammographic views.

^§^For statistical purposes, benign and atypical cases were grouped as “benign” and carcinoma in situ and invasive carcinoma were grouped as “malignant.” Chi-squared tests were conducted.

**Table 4:** Distribution of the diameter of the mammographic lesion of calcifications and final outcome*.

Diameter of lesion of calcifications (mm)^†^	Total *n* = 195^‡^ (% of all lesions)	Benign outcome (*n* = 165)			Malignant outcome (*n* = 30)			Benign versus malignant^§^ *P* value
		Benign *n* = 161	Atypical *n* = 4	Total benign^§^ *n* = 165 (% of this diameter lesion)	In situ carcinoma *n* = 14	Invasive carcinoma *n* = 16	Total malignant^§^ *n* = 30 (% of this diameter lesion)	
0–5	89 (45.64)	81	1	82 (92.1)	7	0	7 (7.9)	<0.0001
6–10	49 (25.1)	40	2	42 (85.7)	4	3	7 (14.3)	<0.0001
11–20	26 (13.3)	18	1	19 (73.1)	1	6	7 (26.9)	0.0186
21–50	22 (11.3)	16	0	16 (72.7)	1	5	6 (27.3)	0.0330
>50	9 (4.6)	6	0	6 (66.7)	1	2	3 (33.3)	0.3173

*Outcome determined at assessment which may include results of FNA, UCNB, VALCS, or open surgical biopsy or surgical treatment pathology whichever is the latter.

^†^The diameter of the area containing calcification was measured on the nonmagnified mammogram demonstrating the largest diameter.

^‡^Lesions without diameter recorded were excluded from data in this table.

^§^ For statistical purposes, benign and atypical cases were grouped as “benign” and carcinoma in situ and invasive carcinoma were grouped as “malignant.” Chi-squared tests were conducted.
